# Preventive breast health care, an embarrassing subject for women with intellectual disability

**DOI:** 10.1177/17446295251326010

**Published:** 2025-03-15

**Authors:** Päivi Adolfsson, Cecilia Arving, Marie Lange

**Affiliations:** Department of Public Health and Caring Science, 8097 Uppsala University, Sweden; Department of Public Health and Caring Science, 8097 Uppsala University, Sweden; Department of Food Studies, Nutrition and Dietetics Uppsala University, Sweden

**Keywords:** breast cancer, disabilities, mammography, reduced health gaps, screening

## Abstract

Women with intellectual disability run an increased risk of dying of breast cancer compared to the general population. There is a clear connection between late detection and low participation in the mammogram screening program. This study aimed to explore the views and experiences of breast health care in women with intellectual disability. Semi-structured interviews were performed individually and in small groups among 11 women with intellectual disability in Sweden. Breast cancer screening is not adapted to women with intellectual disability. Insufficient support and disrespectful treatment can cause ignorance, fear and anxiety. Familiarity with processes and personal networks are examples of improvement in participation. Preventive breast health care is a sensitive topic for many women with intellectual disability that needs to be addressed. Preventive breast health care needs to be more inclusive and better adapted. Personal networks must not determine the possibility of preventive care.

## Introduction

People with intellectual disability experience significantly more health problems compared to the rest of the population. There are indications that this group is not receiving adequate health care. For example, they often do not appear in health examinations and screening programs (Davis et al., 2014; Khan et al., 2024). Life expectancy for people with intellectual disability has increased, but with that, also the number of age-related diseases such as cancer among them (Banda et al., 2024; O’Leary et al., 2018; O'Regan and Drummond 2008).

Breast cancer is the most prevalent type of cancer among women. The incidence of breast cancer among women continues to rise annually, while the mortality rate associated with the disease shows a decline (Sedeta et al., 2023). Some well-known risk factors for breast cancer are age, personal history, family history, age of menarche lower than 11 years, overweight and nulliparity (Howell et al., 2014). These factors correspond well with women with intellectual disability, especially overweight and nulliparity, as problems with obesity are well known (Ranjan et al., 2018), and many women with intellectual disability may be childless (Umb Carlsson, 2021). Many people with disabilities who develop cancer have less chance of recovery than the general population (Cuypers et al., 2022). Women with intellectual disability face a higher risk of death from breast cancer due to late detection. (Ng et al., 2017; Satgé et al., 2014). In Sweden, mortality is five times higher in women with intellectual disability than women in the general population (National Board of Health and Welfare, 2024; Ng et al., 2017).

Screening is a systematic examination to identify people with a condition or disease. Breast cancer screening includes self-examination and mammography. Regular breast checks increase the chances of quickly detecting changes and improving survival odds. Usually, most countries have a breast screening program and mammography is free. Women with intellectual disability are less likely to examine their breasts regularly compared to women in general (Cobigo et al., 2013; Horsbøl et al., 2023; Khan et al., 2024; Xu et al., 2017). These women are at a heightened risk of mortality due to breast cancer, with late detection playing a prominent role (Khan et al., 2024; Ng et al., 2017; Tsitsi et al., 2021).

Swedish organisations (i.e., The Breast Cancer Association and Cancer Fund) encourage women to examine their breasts regularly. About 60 % of breast cancer cases are detected through mammography, with the prognosis consistently improving (National Board of Health and Welfare, 2022). The national breast screening program offers free mammogram screening to all women between 40 and 74 years, every 18 to 24 months, which has reduced mortality by 16–26% (National Board of Health and Welfare, 2023). The provision of free mammogram screening addresses the financial barriers that prevent individuals from participating (National Board of Health and Welfare, 2023).

Women are sent an invitation letter in accordance with a standardised national protocol containing the clinic’s address and a suggested examination time that can be modified. While overall participation in the screening program in Sweden is high (>80%) (Eurostat, 2023), it is considerably lower among marginalized groups among them women with intellectual disability, women born abroad or socioeconomical low groups (National Board of Health and Welfare, 2023). Women with intellectual disabilities are members of a demographic that is widely acknowledged to face a heightened susceptibility to declining health. Hence, it is imperative to shed light on this issue to narrow these health disparities (National Board of Health and Welfare, 2016). Breast cancer patients who are diagnosed with the disease at later stages (3 and 4) have a reduced likelihood of recovery (National Board of Health and Welfare, 2024). In Sweden, mortality in breast cancer is five times higher in women with intellectual disability than in women in general (National Board of Health and Welfare, 2024; Ng et al., 2017).

Numerous factors contribute to the underrepresentation of women with intellectual disability in mammogram screening, including psychological or physical barriers, lack of knowledge and experience or communication problems (Chan et al., 2022). Different barriers are not always exclusive to this particular group, but they can result in significant obstacles due to their intellectual disability (Llewellyn et al., 2011; Wilkinson et al., 2011; Yankaskas et al., 2010). Insufficient research exists on preventive breast health care for women with intellectual disabilities in the Swedish context. This study aimed to explore the views and experiences of breast health care in women with intellectual disability in Sweden.

## Method

The present study is an explorative qualitative study that includes semi-structured individual and group interviews (Patton, 2015). The study has ethical approval from the Swedish Ethical Review Authority: Dnr: 2023-07024-02.

### Study participants

A purposive sample was used to identify study participants (Patton, 2015). The inclusion criteria were women with intellectual disability able and willing to talk orally about breast cancer and mammography. The recruitment process commenced by reaching out to a local branch of the Swedish National Association for People with Intellectual Disability (FUB) in the eastern region of Sweden.With their assistance the researchers got in contact with three different evening leisure activities for people with intellectual disability. Two of them were cafés that were visited by reserarchers several times. During these visits, both women with intellectual disability and supporting staff from residences for people with intellectual disability were contacted. These visits resulted in two individual and a group interview with three persons (n = 5 study participants). On a single occasion, researchers visited a discussion club for young adults. This visit resulted in one group interview with two study participants. Moreover, local FUB activity leaders recruited four additional study participants from other FUB activities. That resulted in one group interview and two individual interviews. Thus, 11 women with intellectual disability participated in the study. All participants were informed about the study orally and with an easy-to-read information letter with accessible and easy-to-understand text and pictures before they signed a informed consent form.

### Data collection

According to the wishes of the study participants and how they wanted to participate, the data collection was conducted through three group interviews and four individual interviews. The interviews were held in different places, depending on the study participants’ choice. At the beginning of each interview, the study participants were informed again about the purpose of the study (orally and in writing). The interview was audio recorded. Anonymity and confidentiality were explained to each participant. Each interview was conducted using a study specific interview guide that included pictures to illustrate a calling letter, the letter containing the screening response and pictures of the screening instrument.

The interview started with some demographic questions followed by questions concerning the participants’ knowledge of breast cancer and mammography. After that, they were asked about their experiences or expectations about screening and whether they wanted somebody to accompany them to the screening. Additionally, they were asked about the screening practices of other women they knew and if they had any suggestions for increasing the attendance of women with intellectual disability.

### Data analysis

All interviews were transcribed verbatim. The transcribed material was analysed inductively with qualitative content analysis inspired by Graneheim and Lundman (2004) to answer the aim of the study, i.e., to explore the views and experiences of breast health care in women with intellectual disability in Sweden. . The current authors read and reread the unit of analysis to understand the content. As the first step of the analysis, the first author sorted the units of analysis to find meaning units that answered the study’s aim. Every meaning unit was given a numeric code to make it easier to see where they belonged in the material. In the second step of the analysis, the first author shortened these meaning units and condensed them without changing their content. Every condensed meaning unit received an individual code that labeled the content of each meaning unit. Numeric codes were also used in these steps. The same numeric code as a meaning unit labeled the condensed meaning unit and its code (Table 1). After that, all authors discussed the whole material and the first two steps openly until a consensus was reached. In the third step, the first author sorted all the codes into different categories, with each category given a preliminary name. At this stage, all meaning units belonging to the codes within each category were combined to describe each category. After that, the other two authors commented on the categorization, and all authors discussed each category to ensure that their content was mutual within each category and exclusive from the other categories. After the three authors agreed, the first author formulated a preliminary analytic text. During this process, two final categories were formed, with the earlier categories becoming subcategories to them. The preliminary names of the subcategories were changed to more descriptive labels. Authentic interview quotations from all seven interviews are included to illustrate and confirm the findings. The study participants’ names in the quotations and in Table 2 are replaced with pseudonyms.

**Table 1.** Presentation of the analyses of the material, from meaning units, condensation and codes to subcategories and categories.

Mening unit	Condentation	Code	Sub-category	Category
Agatha: no, I go for my check-ups, I was there last November. I get invitation letters and it takes like five minutes and then the mammogram is done, but we have cancer in the family so that’s why I think it’s good to check so you know you’re healthy from cancer	I go for check-ups, get invitation letters and it takes five minutes and the mammogram is done, have cancer in the family so I think it’s good to check you’re healthy from cancer	participation in mammography is a health check-up	*Familiarity with the process*	Ways to master my fears for BHC
Cornelia: I don’t want to ask because ---------- if they do it Interviewer: no, you think it’s a bit difficult to ask such questions Cornelia: yes, it is, they probably do, so I don’t know Interviewer: yes, you have to take care of yourself, right? Cornelia: yes of course	Cornelia: I don’t want to ask if they do it Interviewer: you think it’s difficult to ask such questions Cornelia: yes, they probably do, I don’t know Interviewer: yes, you have to take care of yourself? Cornelia: yes	not interfere in other people’s lives	*Sensitive topic to talk about with just anyone*	Makes it difficult for me to follow breast health care

## Findings

Some 11 women participated in the study. As seen from Table 2, six women were <40 years old and therefore were not included in the mammography program. Still, one of these younger women had undergone screening.

**Table 2.** Characteristics of the study participants and participation in study.

	Age	Interview/ Group interview no	Self-examines the breasts	Experience from mammography screening	Knows what breast cancer is
1 Linda	30	Interview 1^a^	Sometimes	Yes	Unsure
2 Sofia	25	Group interview 1,^b^	Sometimes	No	Yes
3 Jasmine	30	Group interview 1,^b^	No	No	Yes
4.Cornelia	70	Interview 2^b^	Sometimes	Yes	Yes
5 Diana	50	Group interview 2 ^c^	Yes	Yes	Yes
6 Irene	38	Group interview 2^c^	Sometimes	No	Yes
7 Natalie	35	Group interview 3^d^	No	No	Yes
8 Vera	56	Group interview 3^d^	No	Yes	Yes
9 Pearl	39	Group interview 3^d^	No	No	Yes
10 Agatha	46	Interview 3^e^	Yes	Yes	Yes
11 Carola	49	Interview 4^d^	No	Yes	No

^a^FUB local.

^b^Local for leisure time evening activities.

^c^University department.

^d^Participant’s resident.

^e^Café.

**Figure 1.**
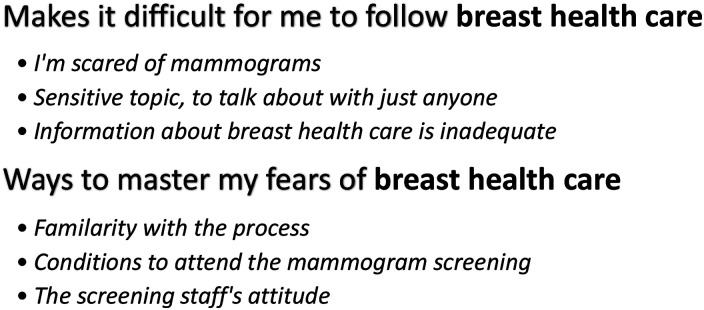
Categories and subcategories.

All study participants who had received the invitation letter also participated in the screening. The participants’ varied experiences have shaped the present findings. The analysis resulted in two categories and six sub-categories. Some subcategories are not representative of all study participants (Figure 1).

### Makes it difficult for me to follow breast health care

Not being aware of the risks of not performing preventive **breast health care** was common among all study participants. Those without experience with mammogram screening stated that they feared undergoing mammogram screening, which was not the case with those with experience. All study participants shared the common practice of discussing breast health care exclusively with their significant others. They had difficulty assimilating and understanding the information they had about mammogram screening.

### I’m scared of mammograms

This subcategory reveals the concerns of those five study participants who had no experience with mammogram screening. Their exclusion was based on their age, with no specific criteria for screening them before the age of 40. Their only source of information about the screening process and experience was through accounts from relatives and supporting staff. Due to their lack of knowledge, they felt apprehensive about being alone in a room with a stranger. They were concerned that undergoing a mammogram screening would lead to feelings of embarrassment and isolation. It was also brought to their attention that the examination might be painful. According to the participants, it would be helpful if someone followed them to the screening.

4.23 Diana: but maybe I'll bring my mom or someone who can be next to me or something like that. I'm so scared (laughs again)

Interviewer: what are you so afraid of, so what is the concern?

Diana: I know, I actually don't know,if it is, … what I feel (laughs again)

The study participants attempted to understand and conceptualize the significance of mammography and breast cancer. However, they associated screening with having breast cancer. They had difficulty understanding why they should undergo screening. They struggled to grasp the concept that mammography serves as a preventive public health measure and does not necessarily indicate a diagnosis of breast cancer. There was apprehension regarding the possibility of a breast cancer diagnosis associated with attending a mammography.

5.28 Natalie: I am just 35 years old

Interviewer 1: no but,

Natalie: I want to continue living

…

Natalie: even if I have Down syndrome, I would get nervous or I would be terrified

(From a group interview with three participants)

### Sensitive topic to talk about with just anyone

The study participants had consistent attitudes about with whom they could discuss their thoughts and experiences regarding mammography. The persons they trusted were typically the closest in their lives (e.g., their mothers). For those without experience with screening, it might be natural that they would refrain from discussing it with their friends, neighbors or even their siblings. However, the study participants also said they seldom heard anyone discuss it.

5.47 Interviewer: but do you know someone who participates in mammography examinations

Pearl: no

Natalie: no, not as far as I know anyway

Interviewer: Do you know anyone who doesn't go then?

Pearl: what?

Interviewer: Do you know anyone who doesn't want to go?

Pearl: no

Interviewer: is it usual that you talk about mammography or breast cancer or cancer at all with friends - - - - or - - -at work - - - here -- when you sit (in the common facilities of the group room)

Complete silence

Interviewer: do you usually talk about something like that (looks at Vera who has own experience of mammography examinations)

Vera: no

Interviewer: do you have any relatives, mother or siblings with whom you talk about this sort of things

Natalie: no

Prolonged silence

(From a group interview with three participants)

Study participants with screening experience stated that they discussed screening with their supporting staff, guardians or mothers, but not others. As Agatha expressed, she was a bit scared before her first screening but was calm after talking to her mother: Agatha: *Mom also explained that it doesn’t hurt.* The mutual attitude of all study participants was that mammography is a sensitive subject they commonly do not discuss with people. They were not curious about other women’s screening customs; instead, they considered it everyone’s own concern.

3.23 Cornelia: I don't want to ask because ---------- if they do it

Interviewer: no, you think it's a bit difficult to ask such questions

Cornelia: yes, it is, they probably do, so I don't know.

Interviewer: yes, you have to take care of yourself, right?

Cornelia: yes of course

### Information about breast health care is inadequate

How to inform women with intellectual disability requires a sensitive approach. All study participants were shown the mainstream invitation letter for mammogram screening during the interview. The perception of the participants was that the invitation letter did not contain adequate information for women with intellectual disabilities. According to them, insufficient information can be the reason some women with intellectual disability do not attend mammogram screening. The participants highlighted the need to tailor the invitation letter for women with intellectual disabilities, considering their unique requirements instead of using a standardized format. The responsibility for improving the information lies with the authorities.

2.44 Jasmine: I think that if there are many people who do not go with IF, which it has turned out to be the case, then it is somewhere, somewhere in that information that they do not understand and there the society should adapt even more, so that becomes even clearer, even better

They implied that, especially before the first visit, the invitation letter should be tangible and contain comprehensive details to ensure recipients understand the screening process clearly. They also considered that the invitation letter has several important crucial facts for the recipients (e.g., information about the clinic’s address and the time booked for their visit). Even so, women with intellectual disability require information which is ease of comprehension, prompting the study participants to propose enhancements to the invitation letter that catered more effectively to the requirements of women with intellectual disability. The participants considered the information should be communicated using easy-to-read, understandable text for persons with intellectual disability. This entails using larger text and shorter sentences compared to the invitation letter they were shown. They recommended using pictures and photographs to illustrate the screening and where it will occur. Incorporating these improvements can render the invitation letter more accessible and understandable to individuals with intellectual disability.

6.36 Agatha: it's good to have pictures to show

Interviewer: yes, that's exactly what I'm thinking, that

Agatha: you'll feel safer going there then, perhaps

The study participants expressed that it is the responsibility of the residence staff, particularly female staff members, to educate women with intellectual disabilities about mammography. According to the participants, outreach activities were needed, including home visits and presenting information about mammography and breast cancer in tangible formats. Furthermore, study visits at the clinic should be arranged as a first step to familiarise those afraid of the screening procedure.

4.88 Irene: ... someone who is working with this can visit and tell them in the group or where they live or something like that, tell the staff and the users and inform them what it's about and so they don't have to feel this fear yes – just as much, but…

### Ways to master my fears of breast health care

The study participants had varying responses to compliance with breast health care measures, self-examination, and mammogram screening. Acquaintance with the procedure enhances understanding and promotes a sense of contentment, resulting in greater compliance. Women with intellectual disability often face challenges when attending mammography. Most of them depend on support to plan and attend mammogram screening. All study participants had an idea and expectations about a mammogram screening. The expectations of the screening staff differed, particularly between individuals with and without prior experiences.

### Familiarity with the process

All women of all ages in Sweden are encouraged to investigate own breasts monthly. It is recommended that the check-up be scheduled in conjunction with showering. Nonetheless, the extent to which the study participants followed the recommendations differed, even after being instructed. As seen in Table 2, some participants occasionally engaged in self-investigation, but only two had the routine to do so regularly.

1.34 Interviewer: do you usually do such examinations and feel like this (the interviewer shows)

Linda: yes,

Interviewer: about which one receives instructions

Linda: I got that when I was younger

Interviewer: but you haven't continued with exams, or have you?

Linda: no

A commonly suggested method to facilitate the process of self-investigation is to incorporate it into a daily activity, such as taking a shower. The level of intellectual determines how tangible the encouragement needs to be. That a person doesn’t follow the recommendations does not mean she does not mind that. Carola explained that it can be enough for a person to wash herself when showering. 7:37 Carola: *no, when I’m washing, wash the body*. According to the study participants, it may be advisable to provide instructions to women with intellectual disabilities and their caregivers on how to perform breast self-examinations.

Despite the low adherence to self-examination of the breasts at home among the study participants, those who were invited to the mammogram screening demonstrated full compliance. The invitation letter they receive seems to remind them of the risks and thus encourages them to attend to avoid unpleasant surprises in the future.

6.1 Agatha: no, I go for my check-ups, I was there last November. I get invitation letters and it takes like five minutes and then the mammogram is done, but we have cancer in the family so that's why I think it's good to check so you know you're healthy from cancer

Those study participants who had experiences with mammogram screening revealed that they had wanted to know how to proceed before their first visit to the clinic. They had felt uncertain about the procedure. According to them, to be more comfortable, they needed information about what would happen when they attended the clinic, specifically, how long the screening would take and how they were expected to act.

1.15 Interviewer: how did you like the mammography?

Linda: it wasn't so fun

Laughter…

Interviewer: did you get enough time so you didn't have to rush?

Linda: it, it was stressful and so...

Interviewer: what would you have wanted to know then

Linda: whether I have it or not.

Based on her previous participation, this study participant asserted that comprehending the entire process can prove challenging without concrete explanations. She expected to get results from the screening if she had breast cancer or not directly during her visit. Knowing the procedure, the clients do not expect to get the screening results directly; they know that a letter with screening results will be mailed to them afterward. Although they were familiar with the entire screening procedure, having to wait for the results from the screening was troublesome for some.

However, the study participants who had attended the screening several times were familiar with the procedure. They appreciated that the screening procedure was swift and efficient. With earlier experiences from screening, they have an understanding of the short screening duration. They are aware that the screening process is swift and devoid of any subsequent pain.

3.10 Interviewer: what's it like when you're going there? Does it feel …

Cornelia: no, it feels, in fact, flat, you have to do it. They flatten them

Interviewer: but it goes by pretty quickly, doesn't it?

Cornelia: yes, yes, yes ---

After a while

Cornelia: as long as it gets done

### Conditions to attend the mammogram screening

The study participants explained that women with intellectual disability often depended on support from other people. Most participants are assigned a guardian to whom the invitation letter is addressed. It is the guardian’s responsibility to relay this information to the participants. They stated the guardians are not responsible for coordinating the visit. The supporting staff is responsible for following the participants if necessary.

5.8 Interviewer: does your guardian usually accompany you or take care of it, so it’ll be done?

Vera: no! She only takes care of my bills

Interviewer: but she calls and says that now is the time

Vera: yes

The study participants revealed that they cannot select the staff member of their preference to accompany them. Instead, the person who will accompany them to the screening is the staff member on duty.

According to the study participants, a positive factor for the attendance of mammography among women with intellectual disabilities is the absence of any cost for the screening. They mentioned that this group had limited finances and therefore would probably think about whether they could afford to attend the screening or if they needed to spend money on something else.

### The screening staff’s attitude

The study participants without experience of breast cancer screening expressed anxiety regarding the screening nurses’ attitudes towards them. Their prior knowledge of various health care screenings and treatments, as well as their encounters with negative behavior from health care professionals, shaped their expectations of staff members at mammography clinics.

2.54 Jasmine: yes, but it's the behavior too, I'm so incredibly disappointed with the way doctors treat people with disabilities - they need more knowledge about ID, because our ID is not visible

I: No, no, no

Sofia: but it's still there

Those with mammogram screening experiences had a positive attitude toward the screening staff. During their conversation, they discussed their appointment experience, including details about the screening process, how to locate the screening section and the staff members they encountered. According to the participants, clear signs inside the hospital helped them find the way to the screening unit. Primarily, the study participants appeared to have a sense of security in the presence of the nurses during the screening procedure. They expressed that the nurses were responsive and sensitive to their needs. The participants were provided with answers to their inquiries, and the nurses offered explanations when necessary.

4.31 Irene: and I say that if it was the first time, I say, “I'm very nervous”, “yes, then we'll take it easy” and not like this, what's the name of the one (nurse) who whines and stresses you out, then we'll take it easy like that, and they show how to stand and how to hold hands and so on and now we take there and then you take from the front and the side and everything so there are no problems.

When the study participants were asked their opinion about whether the gender of screening nurses matters, they responded with varying views. Some, especially those without earlier mammography experiences, hesitated to be treated by a male nurse. In a discussion between one study participant with screening experiences and an aversion towards male nurses and two without such experiences, they agreed about the gender of the screening nurse.

5.31 Interviewer: Vera you who has experience, has there only been female personnel there

Vera: yes

Interviewer: if there was a male nurse

Vera: then I won't take off

everyone laughs

Pearl: me neither

Natalie: me neither

Certain participants believed that the screening nurse’s sex was inconsequential. However, there was a general understanding that it is difficult for everyone to ignore the sex of health care professionals. They pointed out that a first meeting with a male staff member might be embarrassing, but getting used to their treatment is possible.

4.36 Irene: I'm not so afraid of male nurses because I have a doctor who, or a family doctor who is a man, so I, so I don't think about it like that but, maybe a little the first time, but then you get used to it, after a while so

## Discussion

The present qualitative study aimed to explore the views and experiences of breast health care in women with intellectual disability in Sweden. The findings show that screening for breast cancer is not adapted to the needs of women with intellectual disability. Moreover, our findings indicate that women with intellectual disabilities express a desire to gain additional knowledge regarding breast health care to adequately prepare themselves for screening. The participants described the necessity for sufficient support and dignified treatment to oversee mammogram screening. Not receiving adequate support leads to ignorance, unnecessary fear and anxiety about breast health care for these women.

The five study participants who were >40 years old declared they always participated in mammography examinations when invited. Some authors observe that women with intellectual disability exhibit lower rates of participation in mammography examinations compared to women in the general population (Cobigo et al., 2013; Horsbøl et al., 2023; Khan et al., 2024; Olsen et al., 2023; Xu et al., 2017) and that breast cancer in these women is detected at a later stage (National Board of Health and Welfare, 2024; Ng et al., 2017; Satgé et al., 2014). The younger participants in the study experienced feelings of anxiety, embarrassment and abandonment regarding for an unpleasant painful experience in future of mammogram screenings. Nevertheless, they believed receiving support from staff or family members during screening could alleviate these negative emotions. These emotions were observed in earlier studies (Chan et al., 2022; Truesdale-Kennedy et al., 2011; Willis, 2016).

The individuals who had undergone mammography in this study reported experiencing discomfort. Still, the discomfort was transient and did not hinder them from participating in further mammography examinations, which has been confirmed by other studies (Truesdale-Kennedy et al., 2011; Yankaskas et al., 2010). However, anxiety about pain related to mammogram screening is widespread among women in general (Loving et al., 2021). Nevertheless, the screening procedure can elicit differing experiences (Llewellyn et al., 2011). In their scooping review, Ding et al. (2024) discovered that healthcare professionals actively seek diverse strategies to address issues and enhance patients’ mammogram screening experience. Some strategies they mentioned were reducing pain by improving the screening procedure, administering painkillers, and improving patient information (Ding et al., 2024). The absence of tailored information poses challenges for women with intellectual disability in adhering to recommended breast health care practices, such as regular mammography examinations and self-examinations of the breast (Banda et al., 2024; Llewellyn et al., 2011; O'Regan and Drummond, 2008).

The study participants suggested several improvements to the mammogram screening information in the invitation letter. These suggestions included using visually appealing materials such as images, videos, and verbal explanations instead of written text. Women with intellectual disability, frequently accompanied by literacy difficulties, require simplified information. Acquiring appropriate information makes it easier for them to master their fears for breast health care. Moreover, studies show that is important to enhance awareness and expand understanding regarding the benefits of mammography for women with intellectual disability (Llewellyn et al., 2011; Mcilfatrick et al., 2011; Truesdale-Kennedy et al., 2011; Walsh, Hegarty et al., 2022; Weise et al., 2024; Willis 2016). Walsh, Hegarty and colleagues (2022) also pointed out the advantages of focusing on breast awareness rather than breast cancer awareness. Breast awareness occurs during ordinary activities and involves a woman being aware of how her breasts normally look and feel and knowing what changes to expect during her lifetime.

Within the study cohort, there existed a lack of awareness and a failure to comprehend the importance of self-examination in the absence of symptoms. For women with a lower cognitive ability, it can be perceived as abstract and difficult to understand the meaning of preventive care. They often lack an understanding of the mammogram screening process, as evidenced by the studies of Yankaskas and colleagues (2010) and Truesdale-Kennedy and colleagues (2011). Yankaskas et al. (2010) emphasize the need for increased education and information regarding self-examination and mammography among women with intellectual disability, intending to prevent future issues rather than treat current ones.

The present findings show that it was unusual among the study participants to carry out self-examinations of the breasts. The inability of women with intellectual disabilities to perform self-examinations may be attributed to memory deficits or cognitive limitations. For instance, their lack of understanding regarding the signs, risks, and symptoms of breast cancer functions as a barrier to their awareness of this disease. Among the study participants, the diagnosis of cancer in a family member or friend changes their perception of breast cancer from an abstract concept to a tangible reality. Other studies corroborate this finding (Llewellyn et al., 2011; Mcilfatrick et al., 2011; Walsh et al., 2022; Willis, 2016). It is recommended that individuals with intellectual disability receive regular reminders to conduct breast self-examinations and receive personalised health information (Chinn and Homeyard, 2017; Weise et al., 2024).

The study participants stated that mammography and self-examination of the breast are not topics for discussion with anybody. Instead, they suggested people close to them, such as mothers, trusted residential staff or closed friends, could assume the responsibility of discussing mammography or breast self-examination. Conversely, they suggested that emotionally close persons could take the responsibility of discussing mammography or breast self-examination. However, people with intellectual disabilities often have fewer friends than people in general. Social networks, regardless of size, positively affect the quality of life for people with intellectual disability (Umb Carlsson and Adolfsson, 2023). Friends are commonly perceived as individuals with whom one can engage in meaningful and reliable conversations, although maintaining contact with them can present difficulties and be contingent upon support (Callus, 2016; Fulford and Cobigo, 2018; Harrison et al., 2021; Jackson et al., 2024). This could potentially impede the free discourse surrounding these matters and the opportunity to share personal experiences with friends. The study revealed the importance of significant others, particularly mothers, and their role as valued enablers in promoting cancer prevention measures such as mammography. The presence of family and friends is crucial for the study participants to receive support in their participation in breast health care. However, Cuskelly et al. (2006) raised the problem of parents becoming old or otherwise lacking ability and can no longer provide support. Relying on the availability and kindness of relatives for proper preventive care contradicts the notion of equitable treatment (Llewellyn et al., 2011). Family and professional contacts are important (Van Asselt-Goverts et al., 2015). Still, participation in mammography and breast self-examinations should not depend on caring relatives. According to the findings of Arving and Holmstrom (2011), psychosocial nurses played a crucial role in the care of breast cancer patients who lacked a supportive social network. The likelihood of discussions about breasts and mammography is reduced when there are fewer close relatives, especially considering the sensitivity associated with the topic. Willis (2016) suggests that information and promotion of breast health care be provided to women with intellectual disability during their daily activities or within their residences. Engaging in this activity as a collective may foster a more casual approach to breast health care, facilitating open discussions with friends. In addition, residential staff could function as a support for participation. Nonetheless, the study participants highlighted the disadvantage of having a reduced opportunity to choose their companions due to their dependence on the on-duty staff. For example, our participants expressed difficulties accepting male nurses/residential staff, which also seems to vary among female patients (Fitzpatrick et al., 2008). Should participants have a lack of confidence in their staff, it could impede their ability to participate.

Participants in the study highlighted another hindrance to their involvement in mammography examinations, i.e., the perception of negative attitudes exhibited by health care professionals. These negative experiences can jeopardize the willingness of women with intellectual disability to participate in mammogram screening programs. These negative experiences highlight the lack of awareness among healthcare professionals and underscore the necessity for enhanced knowledge and practice to incorporate individuals with intellectual disabilities into the healthcare system. This observation is also evident in other studies ( Chan et al., 2022; Flynn et al., 2015; Llewellyn et al., 2011; Mcilfatrick et al., 2011; Walsh, O’Mahony et al., 2022; Willis, 2016).

## Limitations

The trustworthiness of the present study is discussed in relation to the procedures of Graneheim and Lundman (2004). This study employed a qualitative design that included 11 participants with intellectual disability who expressed their view of breast health care. Through the application of purposeful sampling (Patton, 2015), female individuals with intellectual disabilities who will engage in discussions about breast health care were identified and included in the study, thereby validating its credibility. The sample was small (n = 11) but exhibited heterogeneity. The study participants’ diversity yielded a wide range of personal experiences.

Nevertheless, we aimed to include representatives of women with intellectual disability who declined participation in breast cancer screening despite being invited, but we could not achieve this goal. According to the research findings, discussions regarding breast health care and breast cancer appear to be delicate subjects among women with intellectual disability, potentially impacting their inclination to participate. The research group possesses a wealth of expertise in the field of intellectual disability and their care, as well as cancer care and the research team possesses extensive expertise in qualitative research methods and conducting interview studies, thereby enhancing the study’s credibility. Using a study-specific interview guide with picture illustrations also promotes credibility.

A thorough depiction of the data collection and analysis process was presented to achieve conformability. The findings were validated by employing genuine quotes from the participant interviews. The character of quotes differs depending on the diverse communication abilities of the women. The interviewer needed to use more probing questions for some of the women to ensure that they understood what the woman meant. In addition some quotes are part of a discussions between the participants including the interviewer comments to encourage the discussion. The interviews were conducted in the presence of one, two or three participants. Consequently, according to the findings, the issue under discussion was not determined by the number of participants but rather by their views and experiences with mammogram screening.

This is a qualitative study which limit the generalization of the results. However the inclusion of the study’s context enables readers to evaluate the transferability of the findings by examining the experiences and personal characteristics of the participants. According to Graneheim and Lundman (2004), the richness of the presentation and suitable quotations can benefit that process.

## Conclusion

Preventive breast health care is a sensitive area for women with intellectual. It is imperative that this attitude be altered for the benefit of these women, and it is equally important that their networks acknowledge this. To bridge the health disparities, there is a need for greater inclusivity and better customization of preventive breast health care for women with intellectual disabilities, focusing on information, education and trust. The possibility of preventive care should not be determined by an individual’s network.

## Data Availability

Only the easy-to-read information letter is available. The collected data will not be shared, written in Swedish and the participants were promised that no one else than the research group will have access to the material.*
